# Natural History of Pediatric Low-Grade Glioma Disease - First Multi-State Model Analysis

**DOI:** 10.7150/jca.33463

**Published:** 2019-10-17

**Authors:** Anna-Maria Goebel, Astrid K. Gnekow, Daniela Kandels, Olaf Witt, Rene Schmidt, Pablo Hernáiz Driever

**Affiliations:** 1 Charité-Universitätsmedizin Berlin, Corporate member of Freie Universität Berlin, Humboldt-Universität zu Berlin, and Berlin Institute of Health, Department of Pediatric Oncology/Hematology, Berlin, Germany; 2 Augsburg University Hospital, SIOP-LGG central study registry, Swabian Children's Cancer Center, Augsburg, Germany; 3 Heidelberg University Hospital, Department of Pediatric Hematology and Oncology, Heidelberg, Germany; 4 German Cancer Research Center (DKFZ) and German Consortium for Translational Cancer Research (DKTK), Heidelberg, Germany; 5 Hopp Children's Cancer Center at the NCT Heidelberg (KiTZ), Heidelberg, Germany; 6 University of Muenster, Institute of Biostatistics and Clinical Research, Muenster, Germany

**Keywords:** pediatric low-grade glioma, multi-state model, survival, multiple interventions, chronic progressive disease

## Abstract

**Background**: Pediatric low-grade glioma [PLGG] is often a chronic progressive disease requiring multiple treatments, i.e. surgery, chemotherapy and irradiation. The multi-state model [MSM] allows an extended analysis of disease-states, that patients may undergo, incorporating competing risks over the course of time.

**Purpose**: We studied disease-state-probabilities of the German SIOP-LGG 2004 cohort from the initial state “*diagnosis*” to the final state “*death”.* Transient “*disease-states”* incorporated successive surgical and non-surgical treatments. We evaluated clinical risk factors for highly progressive disease requiring multiple interventions and death.

**Results**: We identified 22 states within 1587 patients (median follow-up 6.3 years). For robust statistical calculation, we reduced the model to 7 states and eventually to three levels of disease-progressiveness: non, low and highly progressive. Five years after diagnosis state-probabilities were: 0.11 no therapy, 0.49 one and 0.11 two or more surgeries only, 0.19 one and 0.06 two or more non-surgical interventions with or without prior surgery. At this time point higher probability for highly progressive disease was found in infants (0.30), supratentorial-midline location (0.17) and diffuse astrocytoma WHO-grade II (0.12). Neurofibromatosis type-1 patients were most likely not to be treated (0.36) or to have received only non-surgical therapy (0.45). Two years after diagnosis 3-year predictions for highly progressive disease and death increased with the number of interventions patients underwent in the first 2 years after diagnosis.

**Conclusion**: In this first MSM analysis we delineated a refined description of PLGG disease course over time, identifying three levels of progressiveness. Growth behavior in the first two years predicted future progressiveness and death.

## 1. Introduction

Pediatric low-grade glioma [PLGG] are a heterogeneous group of World Health Organization [WHO] grade I and II brain tumors^1^ that represent the most frequent solid primary CNS tumors in the pediatric age group^2^. As 10 to 20 year overall survival [OS] ranges between 80% and 90% the prognosis is generally favorable^2^-^7^. For many patients, a single surgical approach is curative^7^,^8^. In contrast, a relevant number of patients suffer from chronic progressive disease^9^-^12^ requiring multiple and often multimodal interventions^3^,^11^. Each adjuvant therapy, even surgical therapy-only, harbor a relevant risk for increased long-term sequelae such as cognitive deficits, blindness, hearing-loss, hormonal disturbance, or obesity many years after onset of disease^3^,^13^,^14^.

Various studies identified clinical risk factors for poor event- and progression-free survival after first non-surgical treatment such as age < 1 year at diagnosis^5^,^15^-^17^, diencephalic syndrome and/or tumor dissemination at diagnosis^5^,^16^,^18^, diffuse glioma WHO grade II histology^5^,^16^,^17^, location in the supratentorial midline [SML]^5^,^17^, the diencephalon, the spinal cord or the brainstem^3^, thalamic tumor site^15^, incomplete resection^3^,^15^-^17^ or no surgery^3^, tumor size > 3 square-centimeter^15^,^19^, and possibly age > 8 years^16^. While these studies focused on first events and progression after the first non-surgical treatment, clinical outcome and risk factors for multiple interventions, i.e. chronic progressive PLGG disease-forms, have scarcely been addressed so far^12^,^20^.

Classic survival analysis does not discriminate competing risks. Event-probability and influence of cofactors onto occurrence of one event may be rather coarsely estimated when ignoring presence of further possible events with the Kaplan-Meier estimator. Considering only one start and one endpoint limits the depiction of chronic progressive disease with its possible necessity for multiple interventions and the potentially associated risk for deterioration of quality of survival^21^-^24^.

Based on the semiparametric regression model described by Cox^25^, Aalen and Johansen developed a model to estimate transition-probabilities between a finite number of states of non-homogenous Markov chains^26^. In 1986 Kay^27^ introduced a method to analyze survival-time incorporating disease-states and cofactors with a Markov Model. Since then, different multi-state model [MSM] types have been developed to enable a more detailed analysis of the course of chronic diseases and estimations of future events^28^. The recent introduction of easier-to-use software such as the R-packages “*mstate”*^29^ facilitated wider use of MSM analysis in clinical oncology and hematology^30^-^33^. Apart from one single-center study by Zeidner et al.^33^ with a mixed cohort of 71 pediatric and adult patients with chronic myeloid leukemia, none of the pediatric cancer diseases have been analyzed using this model so far. In the field of neuro-oncology the MSM has not been applied at all. Yet, the MSM is especially useful for the application to chronic disease as it harbors several advantages: (i) It allows inclusion of more than 2 time points for survival analysis including competing events for which patients may be at risk. (ii) It describes development of disease over time. (iii) It includes the influence of subsequent events and treatment interventions at different time points onto the further course of disease. (iv) It evaluates more the prognostic covariates that may influence different stages of disease at different time points^24^,^34^-^36^.

Due to the protracted disease-evolution, a more detailed analysis of PLGG is warranted to delineate growth characteristics and dynamic influence of multiple and multimodal treatments, i.e. non-primary events as a result of chronic progressive disease activity. An early identification of patients at risk for highly progressive disease requiring multiple interventions is essential. This may improve future treatment planning and risk estimation for progression as well as brain damage at certain disease-states^24^,^29^,^37^. PLGG are considered a cohort of disease entities characterized by a single canonical pathologic activation of the mitogen-activated protein kinase [MAPK] signaling pathway by various genetic alterations^38^,^39^. Unfortunately, as recruiting of patients started far before these key molecular findings were revealed, molecular information was not available for this MSM study.

So far, only one study with a PLGG-cohort incorporated analysis of competing risks but only for cause of death^4^. This is the very first MSM analysis of a population-based cohort of PLGG patients. Our aim was to provide a unique MSM of patients' progress with transitions to different states beyond the classical survival analysis on the base of recent advances in PLGG treatment strategy. We expected to delineate a more extended and refined course of PLGG disease, to verify previously identified subgroups with less interventions and superior OS, as well as risk groups for highly progressive disease and death. Finally, our aim was to enable a predictive model for future growth behavior and probability for death considering the course of PLGG disease in the first two years after diagnosis. We expect the MSM to facilitate evidence-based decision making in the management of even chronic progressive PLGG in the future and serve as an illustrative basis for prognosis and outcome when discussing with families.

## 2. Patients and Methods

### 2.1 Participants cohort

This multi-state analysis of pediatric patients with LGG included 1587 PLGG patients from Germany, registered in the International Society of Pediatric Oncology - Low-Grade Glioma [SIOP-LGG] 2004 study. SIOP-LGG 2004 was a prospective, multinational intervention-study active from April 1^st^, 2004 to March 31^st^, 2012 for pediatric patients with grade I and II gliomas (according the WHO classifications of 2000 and 2007^40^,^41^) aged 18 years and younger at diagnosis. If possible, patients received the first surgery for tumor resection or diagnostic biopsy and were observed thereafter. Patients with neurofibromatosis type 1 [NF-1] and hypothalamic/visual pathway glioma and children without NF-1, whose tumor showed unequivocal contiguous involvement of the visual pathways were accepted without biopsy. The term “observation” included possible further surgical interventions (e.g. in case of radiologic progressive disease [PD]). Following incomplete resection or radiological diagnosis only, observation was recommended as long as there was no radiologic tumor growth and the patient did not suffer from significant tumor-related symptoms. The definition for “surgical intervention” in our analysis involved any extent of tumor resection as well as diagnostic biopsy, while surgery for other reasons, i.e. shunting procedures for reduction of increased intracranial pressure, was not included. In case of radiologic PD and/or severe or progressive clinical symptoms, patients were stratified to receive non-surgical intervention, i.e. primary chemotherapy [ChT] for children younger than 8 years. For children of 8 years and older, radiotherapy [RT] or ChT could be applied (physician's choice). Patients with NF-1 received standard ChT, i.e. 85 weeks of vincristine and carboplatin [VC]. The protocol included randomization of standard VC versus an intensified regimen with additional etoposide [VCE] for non-NF-1 patients. Central review was performed for histology and imaging^16^. After their first treatment, patients were followed. In case of further need of intervention, i.e. PD or threatening clinical event, patients received further surgical or non-surgical therapy (ChT or RT) at the discretion of the local oncology team.

### 2.2 Statistical analysis/Multi-state analysis

**Figure 1:**
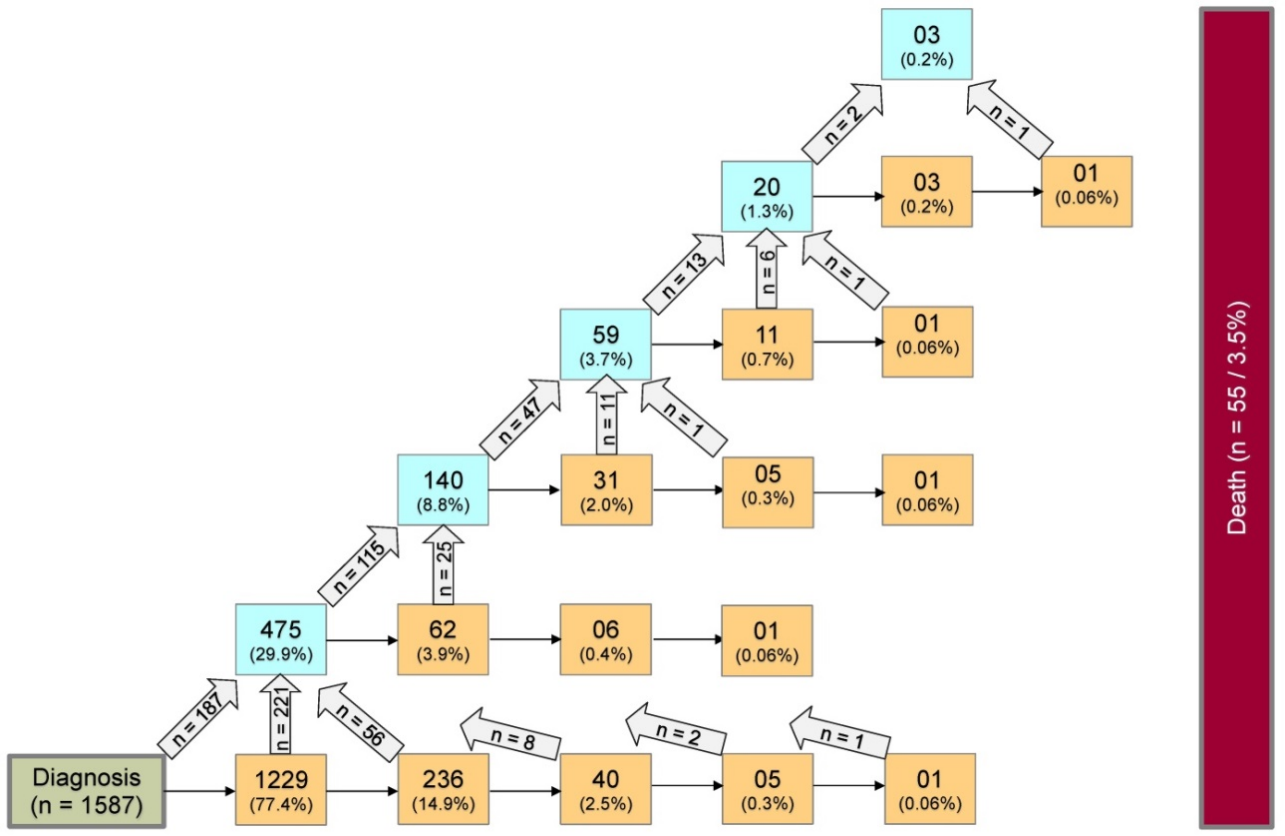
** Whole multi-state model for PLGG:** Complete disease development of whole group of patients with PLGG. Number of states: 22; Number of transitions including those to absorbing state “*death*”: 44; Possible transitions: 56. The numbers in the boxes (states) correspond to the number of patients who had ever reached the corresponding state. The numbers in the arrows represent the number of transitions of patients from one state to the other. Number of transitions in the horizontal plane correspond to the number in each box to the right of the transition and were not specified for reasons of clarity. The number of transitions to death is not shown for reasons of clarity.

**Figure 2:**
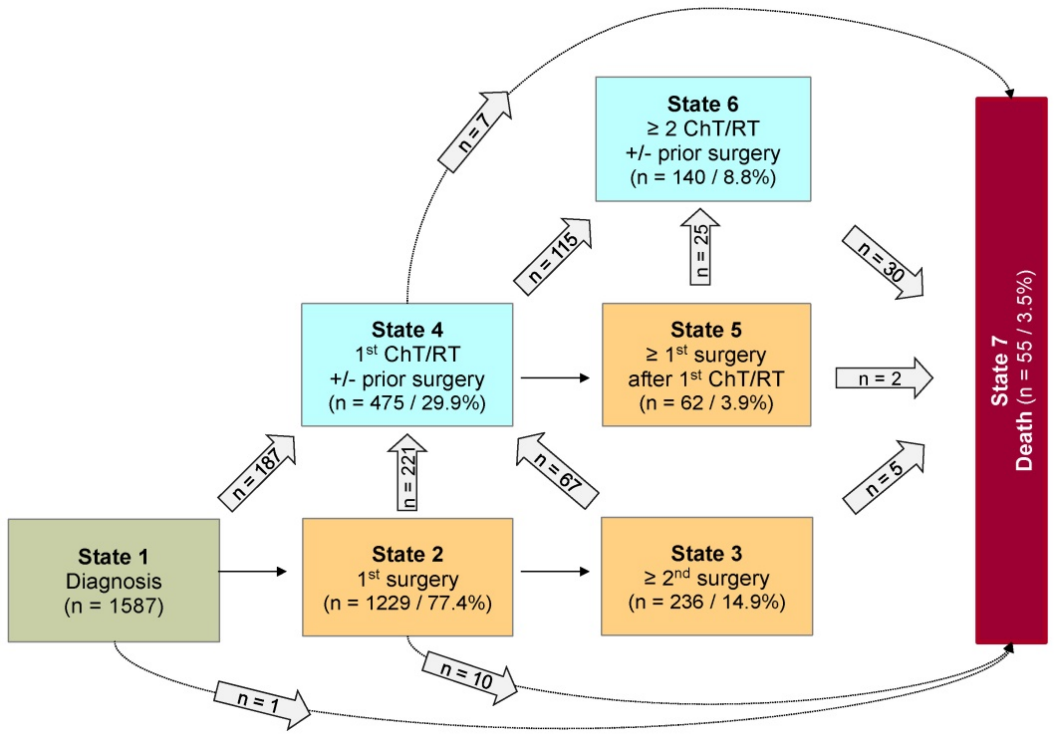
** Seven-state model for patients with PLGG.** Seven-state MSM, used for further calculations. Number of states: 7; Number of transitions: 14. The numbers in the boxes (states) correspond to the number of patients who had ever reached the corresponding state. The numbers in the arrows represent the number of transitions of patients from one state to the other. Number of transitions in the horizontal plane correspond to the number in each box to the right of the transition and were not specified for reasons of clarity. Levels of progressiveness: non-progressive (states 1 and 2), low progressive (states 3 and 4) and highly progressive (states 5 and 6) PLGG. ChT: chemotherapy. RT: radiotherapy**.**

Analyses were done using the statistical software R 3.4.2^42^. Categorical data were summarized as absolute and relative frequency. Metric data were summarized by median, minimum, and maximum. Multi-state analysis was performed to analyze the probabilities and risk factors for non, low, and highly progressive disease requiring multiple interventions following diagnosis, as well as to develop a prediction model with starting point 2 years after diagnosis. For every patient, the outcome of interest was the types of intervention over time, differentiated into surgical and non-surgical intervention (ChT or RT). Accordingly, a Markovian MSM with the clock-forward approach and unidirectional arrows was used. Time was measured in years from diagnosis. Supplemental Figure S1 displays the schematic model of possible states and transitions used for first data structuring. The sequences of observed multiple interventions are summarized in Figure 1. For the multi-state analysis, however, a coarsened model based on only 7 states was considered in which each state was occupied by a sufficient number of patients (n ≥ 60). Figure 2 illustrates this underlying seven-state model. The arrows indicate the directions in which transitions were possible. At any given point throughout the observation period, a patient was classified to be in one of 7 states comprising the initial state “*diagnosis*” (state 1), five transient “*disease-states”* (state 2-6) and the absorbing state “*death of any cause”* (state 7*)*. PLGG histology was centrally confirmed and neuroimaging was centrally assessed at diagnosis, upon progression, and/or following surgical and non-surgical interventions. A patient without any adjuvant therapy so far, could move from state 1 to 2 and from state 2 to 3 at the time of the first and second surgery, respectively. Third and further surgeries as well as 3^rd^ and further adjuvant therapies are not detailed in Figure 2 due to small occupation numbers (< 60 patients per state). At the start of the first adjuvant therapy (ChT or RT), patients moved directly from state 1, 2, or 3 to state 4. A patient with only one adjuvant therapy so far, could move from state 4 to state 5 if a surgery followed first adjuvant therapy. At the start of a second adjuvant therapy, patients moved from state 4 or 5 to state 6. The absorbing state “*death of any cause”* could be reached instantaneously from any other state. The multi-state methodology allowed us obtaining the probabilities for combinations of interventions by defined risk factors during the course of time. State-probabilities at certain time points for the whole cohort and subgroups with at least 40 patients per group were obtained from the Aalen-Johansen estimate^26^ calculated by the “*mstate*” R-package^29^. State-probabilities were based on a transition-specific Cox model. For better inter-study comparability of our results we chose identical classification of subgroups as stated in the preceding HIT-LGG 1996 study^5^ and confirmed in the SIOP-LGG 2004 trial for non-NF-1-patients receiving ChT^16^. Cofactors for analysis of state-specific risk groups and influences were as follows: age group according to age at diagnosis (< 1 year, 1-7^11/12^ years, ≥ 8 years), sex, main histology, main location, and NF-1 status.

We did not analyze subgroups with patient numbers below 40 per group, i.e. disseminated tumor, oligodendroglioma WHO grade II, oligoastrocytoma WHO grade II, Neurofibromatosis type 2 [NF-2] as well as Tuberous Sclerosis Complex [TSC]. Moreover, we refrained from further state-differentiation, i.e. ChT, RT or type of ChT, as stratification for 1^st^ adjuvant therapy and physician's choice on further adjuvant treatment would not allow robust statistical conclusions of later disease course.

**Figure 3:**
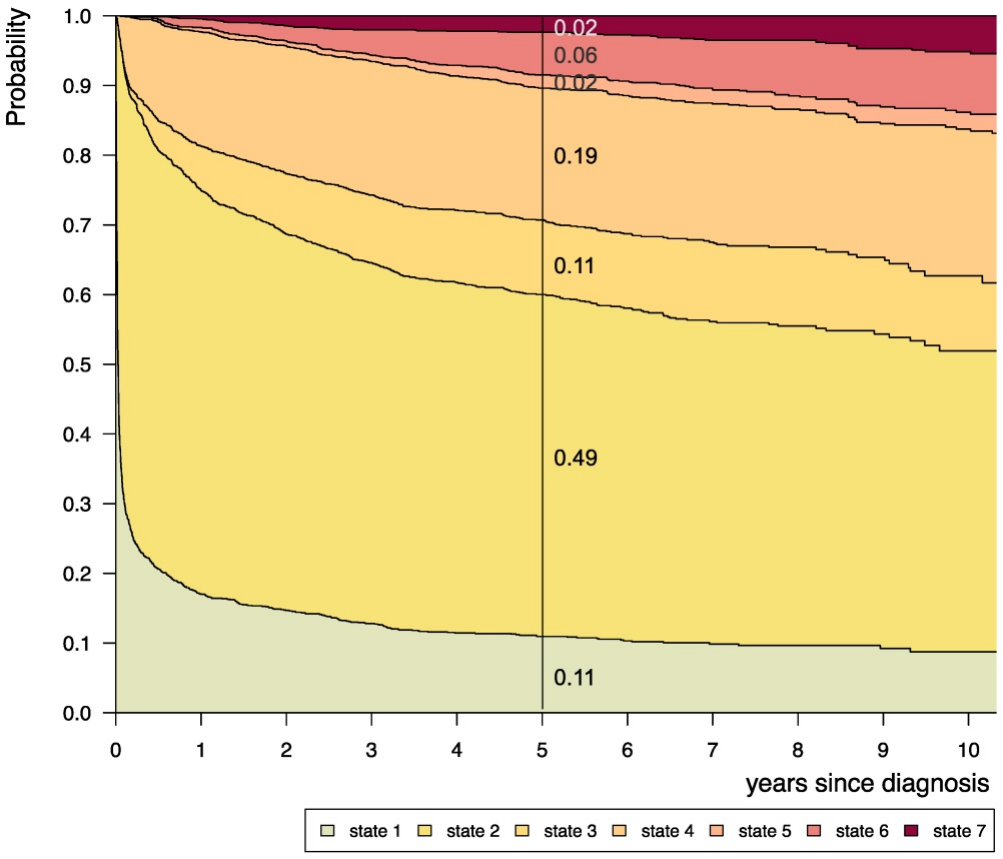
** Disease-state development for whole group of patients from Germany (n=1587) with PLGG.** Stacked-plot of disease-state development over first 10 years after diagnosis for the whole group of patients with PLGG, starting from date of diagnosis. State-probabilities derived from the multistate model of Figure 2. Exact numbers given for state-probabilities at time point 5 years after diagnosis. State 1: no intervention. State 2: first surgery without prior adjuvant therapy. State 3: two or more surgeries without prior adjuvant therapy. State 4: first adjuvant therapy. State 5: one or more surgeries after first adjuvant therapy. State 6: two or more adjuvant therapies. State 7: death of any cause. Levels of progressiveness: non-progressive (states 1 and 2), low progressive (states 3 and 4), and highly progressive (states 5 and 6) PLGG.

**Figure 4:**
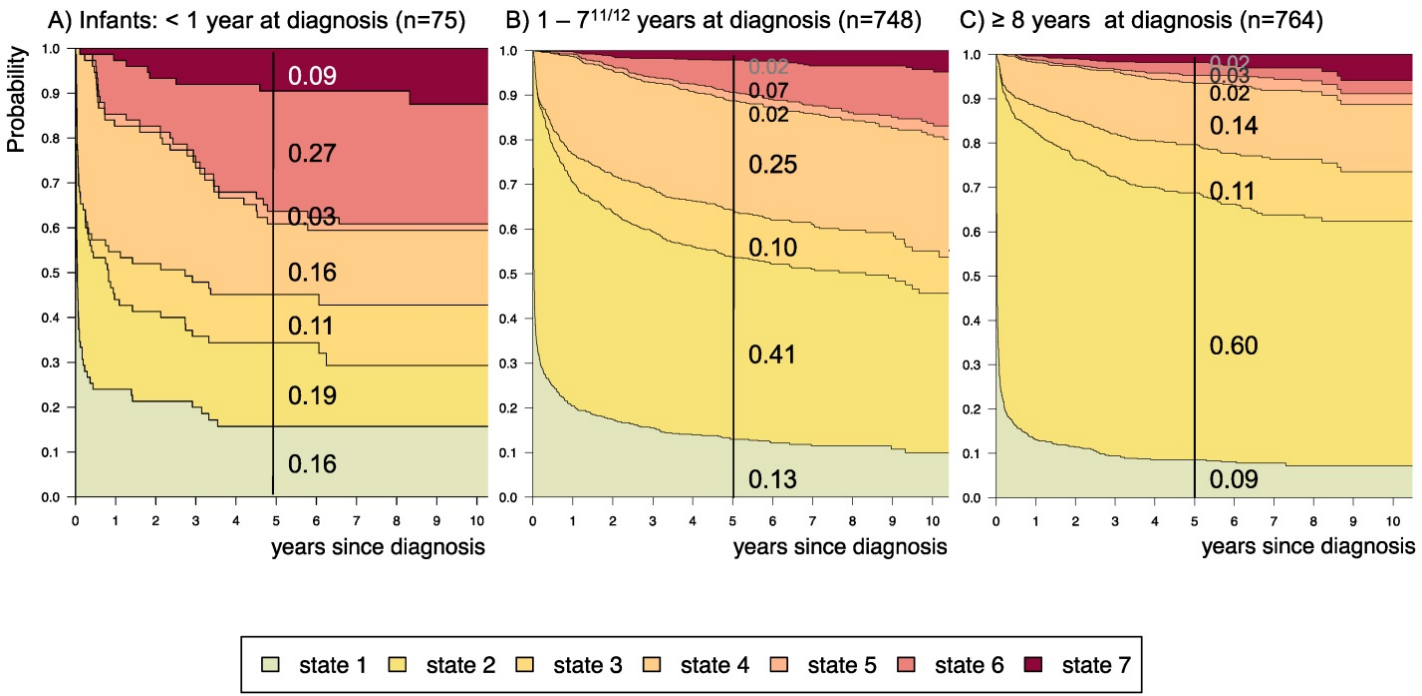
** Disease-state development for patients with PLGG by subgroup *age-group.***Stacked-plot of disease-state development over first 10 years after diagnosis for the subgroup *age-group*, starting from date of diagnosis: (A) Infant-age < 1 year at diagnosis, (B) 1-7^11/12^ years at diagnosis, and (C) ≥ 8 years at diagnosis. State-probabilities derived from the multistate model of Figure 2. Exact numbers given for state-probabilities at time point 5 years after diagnosis. State 1: no intervention. State 2: first surgery without prior adjuvant therapy. State 3: two or more surgeries without prior adjuvant therapy. State 4: first adjuvant therapy. State 5: one or more surgeries after first adjuvant therapy. State 6: two or more adjuvant therapies. State 7: death of any cause. Levels of progressiveness: non-progressive (states 1 and 2), low progressive (states 3 and 4), and highly progressive (states 5 and 6) PLGG.

**Figure 5:**
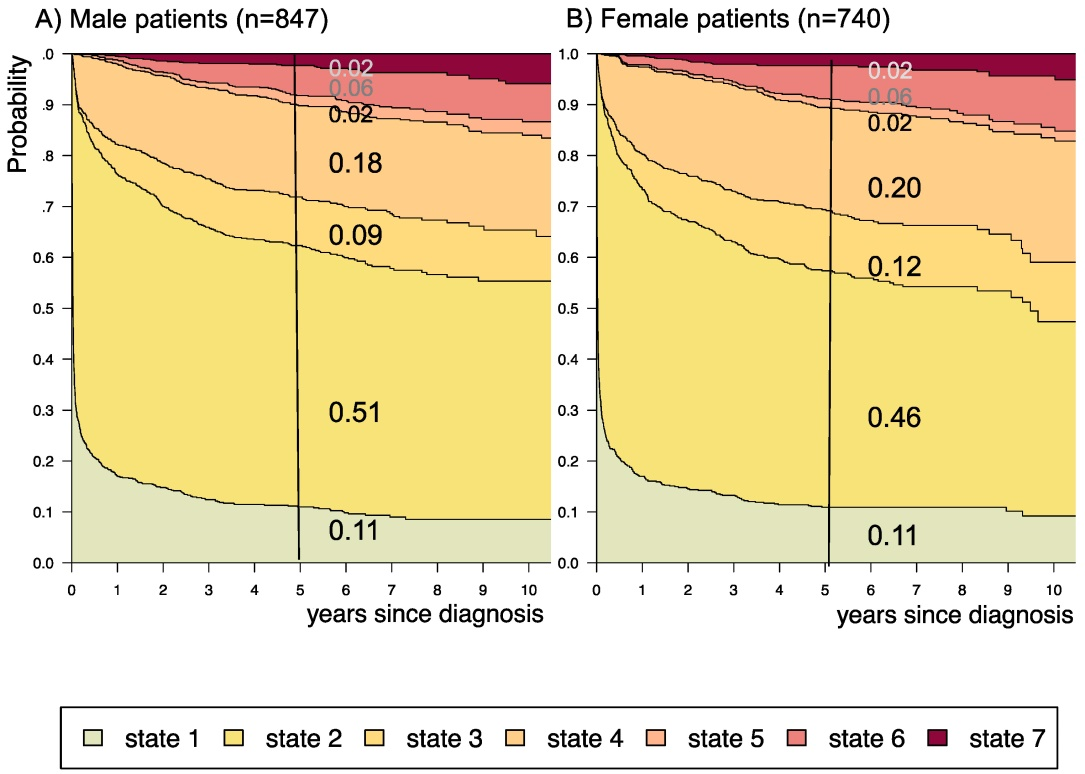
** Disease-state development for patients with PLGG by subgroup *sex.*** Stacked-plot of disease-state development over first 10 years after diagnosis for the subgroup *sex*, starting from date of diagnosis: (A) Male patients and (B) Female patients. State-probabilities derived from the multistate model of Figure 2. Exact numbers given for state-probabilities at time point 5 years after diagnosis. State 1: no intervention. State 2: first surgery without prior adjuvant therapy. State 3: two or more surgeries without prior adjuvant therapy. State 4: first adjuvant therapy. State 5: one or more surgeries after first adjuvant therapy. State 6: two or more adjuvant therapies. State 7: death of any cause. Levels of progressiveness: non-progressive (states 1 and 2), low progressive (states 3 and 4), and highly progressive (states 5 and 6) PLGG.

**Figure 6:**
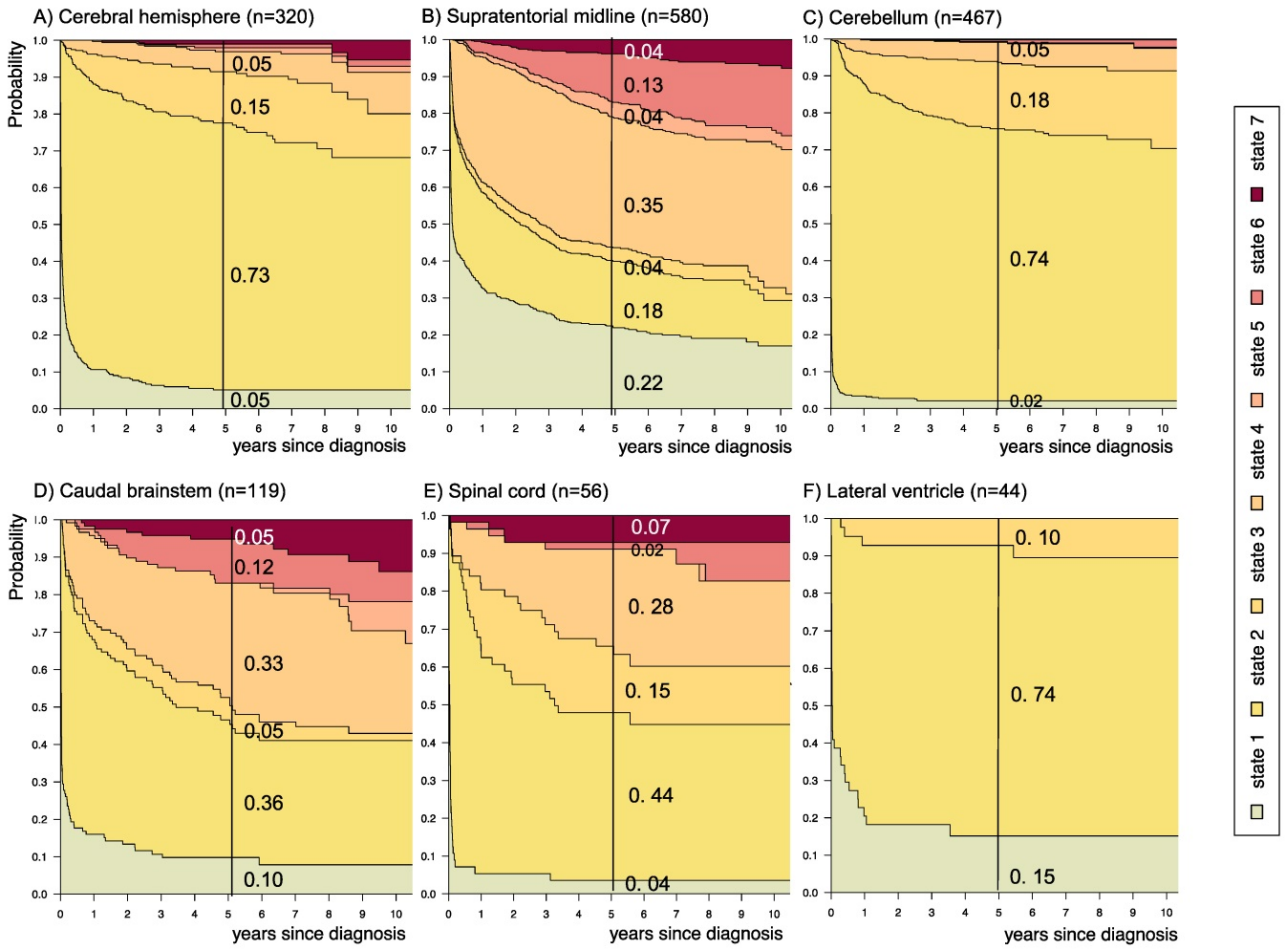
** Disease-state development for patients with PLGG by subgroup *main location.*** Stacked-plot of disease-state development over first 10 years after diagnosis for the subgroup *main location*, starting from date of diagnosis: (A) Cerebral hemisphere, (B) Supratentorial midline, (C) Cerebellum, (D) Caudal brainstem, (E) Spinal cord, and (F) Lateral ventricle. State-probabilities derived from the multistate model of Figure 2. Exact numbers given for state-probabilities at time point 5 years after diagnosis. State 1: no intervention. State 2: first surgery without prior adjuvant therapy. State 3: two or more surgeries without prior adjuvant therapy. State 4: first adjuvant therapy. State 5: one or more surgeries after first adjuvant therapy. State 6: two or more adjuvant therapies. State 7: death of any cause. Levels of progressiveness: non-progressive (states 1 and 2), low progressive (states 3 and 4), and highly progressive (states 5 and 6) PLGG.

**Figure 7:**
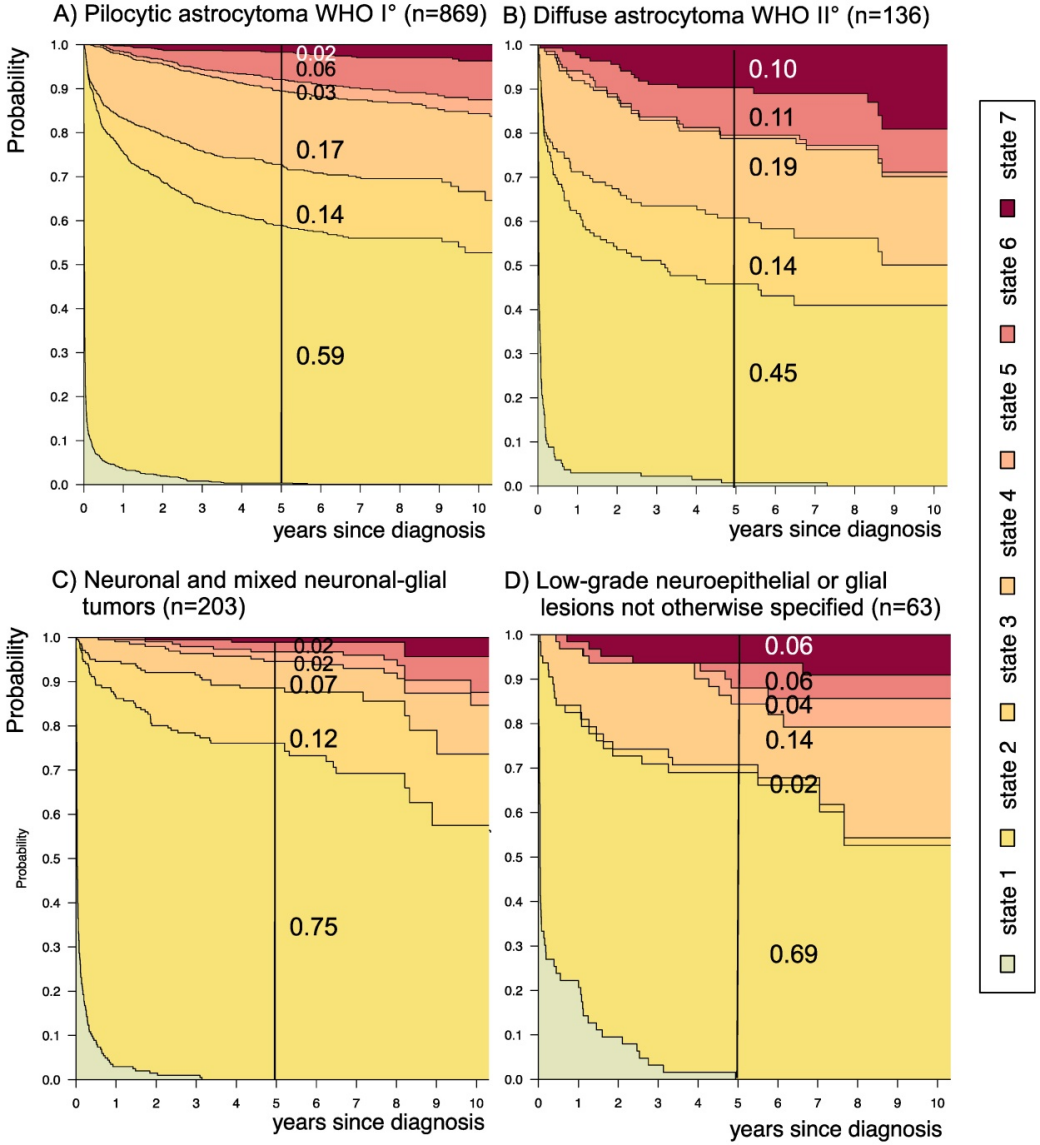
** Disease-state development for patients with PLGG by subgroup *main histology.***Stacked-plot of disease-state development over first 10 years after diagnosis for the subgroup *main histology*, starting from date of diagnosis: (A) Pilocytic astrocytoma WHO grade I, (B) Diffuse astrocytoma WHO grade II, (C) Neuronal and mixed neuronal-glial tumors, and (D) Low-grade neuroepithelial or glial lesions not otherwise specified (LGG-NOS). State-probabilities derived from the multistate model of Figure 2. Exact numbers given for state-probabilities at time point 5 years after diagnosis. State 1: no intervention. State 2: first surgery without prior adjuvant therapy. State 3: two or more surgeries without prior adjuvant therapy. State 4: first adjuvant therapy. State 5: one or more surgeries after first adjuvant therapy. State 6: two or more adjuvant therapies. State 7: death of any cause. Levels of progressiveness: non-progressive (states 1 and 2), low progressive (states 3 and 4), and highly progressive (states 5 and 6) PLGG.

**Figure 8:**
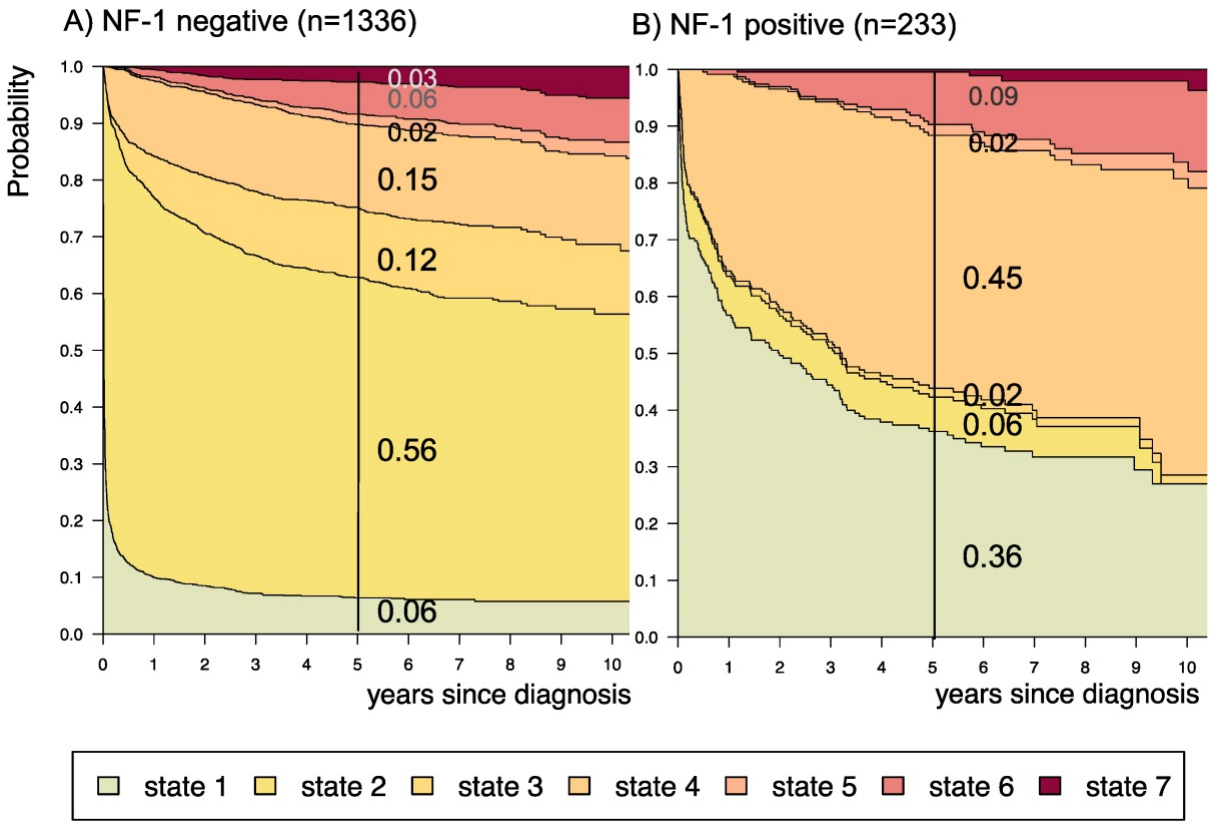
** Disease-state development for patients with PLGG by subgroup *neurofibromatosis type 1 [NF-1] status.*** Stacked-plot of disease-state development over first 10 years after diagnosis for the subgroup *NF-1 status*, starting from date of diagnosis: (A) NF-1 negative and (B) NF-1 positive. State-probabilities derived from the multistate model of Figure 2. Exact numbers given for state-probabilities at time point 5 years after diagnosis. State 1: no intervention. State 2: first surgery without prior adjuvant therapy. State 3: two or more surgeries without prior adjuvant therapy. State 4: first adjuvant therapy. State 5: one or more surgeries after first adjuvant therapy. State 6: two or more adjuvant therapies. State 7: death of any cause. Levels of progressiveness: non-progressive (states 1 and 2), low progressive (states 3 and 4), and highly progressive (states 5 and 6) PLGG.

**Figure 9:**
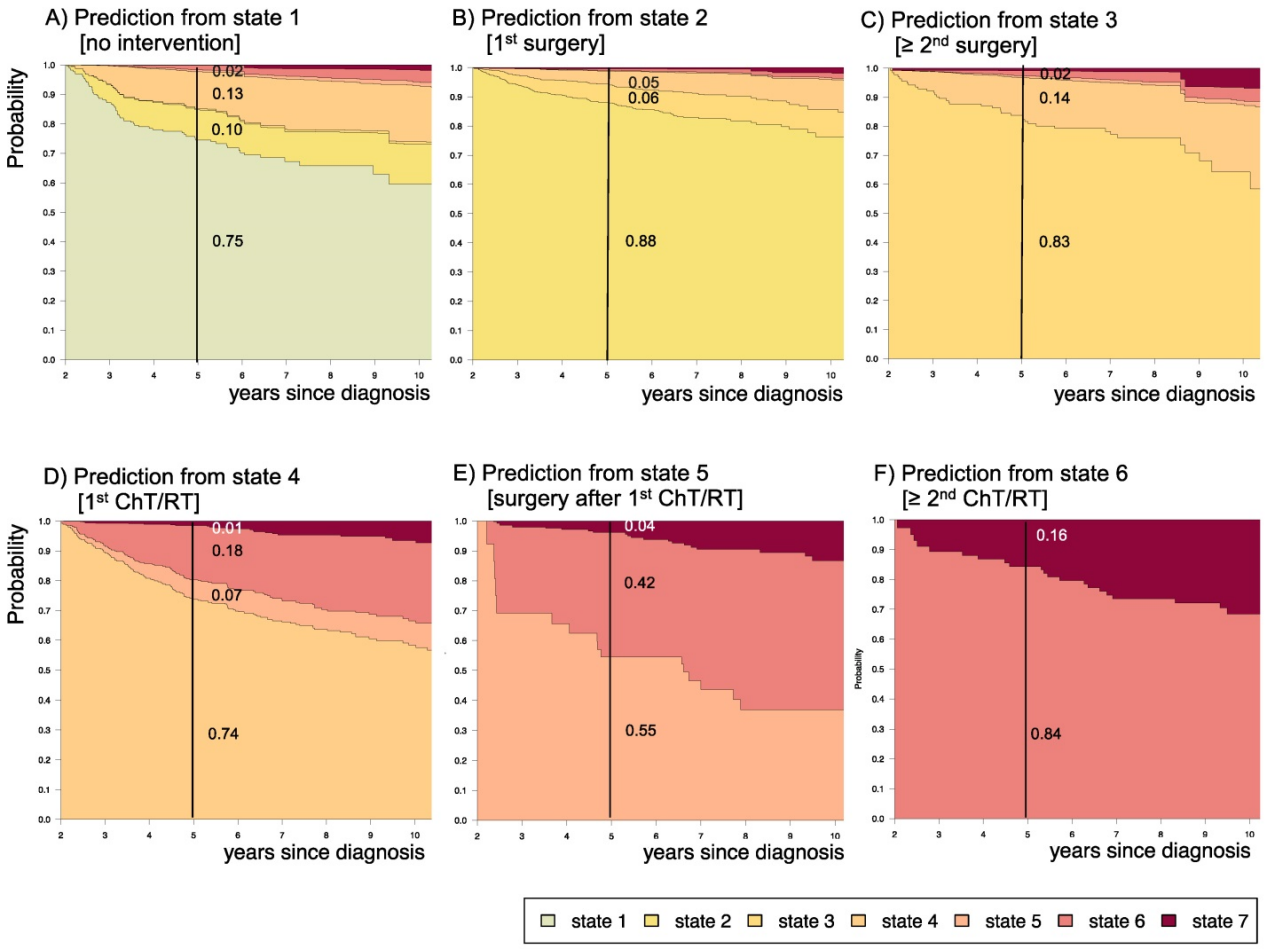
** Impact of state at 2 years after diagnosis on prognosis for the following 3 years.** Stacked-plot of disease-state development from 2 to 10 years after diagnosis for the whole group of patients with PLGG, starting from different states: A) State 1: no intervention, B) State 2: first surgery without prior adjuvant therapy, C) State 3: two or more surgeries without prior adjuvant therapy, D) State 4: first adjuvant therapy, E) State 5: one or more surgeries after first adjuvant therapy, F) State 6: two or more adjuvant therapies. State-probabilities derived from the multistate model of Figure 2. Exact numbers given for state-probabilities at time point 5 years after diagnosis. State 7: death of any cause. Levels of progressiveness: non-progressive (states 1 and 2), low progressive (states 3 and 4), and highly progressive (states 5 and 6) PLGG.

Stacked-plot figures were produced with the “*mstate*” R-package^29^ and are shown with state-probabilities 5 years after diagnosis starting (i) from state 1 at time of diagnosis (Figure 3-8) or (ii) from different states at 2 years after diagnosis (Figure 9). The starting point “*2 years after diagnosis*” was chosen from a clinical point of view, since ChT lasts approximately 1.6 years and patients who received their first adjuvant therapy shortly after diagnosis still had the possibility of a state-transition. Figures 4-8 show the state-probabilities for different subgroups, i.e. age group, sex, location, histology and NF-1 status. Since median observation time has not yet reached 10 years, we cropped all figures at 10 years after diagnosis. We additionally evaluated clinical risk factors that are possibly associated with a higher probability to require multiple interventions or more intense treatment, i.e. any intervention beyond diagnosis and 1^st^ surgery (states 1 and 2). From a clinical point of view we graded progressive disease by the number of subsequent interventions received at the time point of analysis into (i) “non-progressive PLGG” (states 1 and 2) (ii) “low progressive PLGG” including 1^st^ adjuvant therapy or at least a 2^nd^ surgery without prior adjuvant therapy (states 3 and 4) and (iii) “highly progressive PLGG” including interventions beyond 1^st^ adjuvant therapy (states 5 and 6). The analysis was considered as exploratory. Accordingly, no adjustment for multiple testing was done and p-values are not given.

## 3. Results

### 3.1 Demographic and clinical characteristics of cohort

Table 1 summarizes demographic and clinical characteristics of our cohort. We identified 1587 patients with primary diagnosis PLGG treated in Germany from the SIOP-LGG 2004 database and followed them up until April 27^th^, 2016. Median follow-up was 6.3 years. Five years after diagnosis, 36 patients had deceased and 10 tumors had transformed to high-grade glioma. Throughout the observation time we identified 22 disease states and 44 transitions between states, displayed in Figure 1:

- 1229/236/40/5/1 patients received 1/2/3/4/5 surgical interventions without prior adjuvant therapy, respectively.
- 475/140/59/20/3 patients received 1/2/3/4/5 adjuvant therapies with or without prior surgical intervention, respectively.
- 27 transformed to high-grade histology.
- 55 patients deceased.

### 3.2 Multi-state analysis of the whole group of patients with PLGG

Considering only transition-states with at least 60 patients, we summarized 7 states with 14 transitions for the final model (Figure 2). Figure 3 visualizes state-probabilities for the whole group up to an observation time of 10 years. Supplemental table S2 indicates development of state-probabilities and Aalen standard errors [SE] for the first 10 years after diagnosis. Five years after diagnosis the highest probability for the whole cohort was to have received one surgical intervention only (0.49). At this same time point probability for 1^st^ adjuvant therapy was 0.19. We found identical probabilities to have had two or more surgical interventions without prior adjuvant therapy (0.11), and not to have received any treatment at all (0.11). Probability for low progressive PLGG (states 3 and 4) was 0.30 and for highly progressive PLGG (states 5 and 6) 0.08. Probability for death 5 years after diagnosis was 0.02.

### 3.3 Multi-state analysis for probable risk factors

Supplemental table S3 displays 5-year state-probability and Aalen SE for clinical subgroups with at least 40 PLGG-patients. Figures 4-8 show state-probabilities over 10 years after diagnosis for these subgroups. We found a higher 5-year-probability to decease for infants (0.09), patients with tumor location in the spinal cord (0.07), and astrocytic tumors WHO grade II (0.10).

The probability to harbor highly progressive disease was especially high for infants (0.30) with a noticeably higher probability for 2 or more adjuvant therapies (0.27). We found no relevant differences in state-probability for highly progressive PLGG concerning sex. Compared to patients with other tumor locations, those with a SML-glioma showed a high probability to need one (0.35) or at least two adjuvant therapies (0.13) and the highest 5-year-probability for highly progressive disease (0.17). Nevertheless, the probability of patients with a SML-PLGG to stay untreated for at least 5 years after diagnosis still was 0.22. Concerning histology, astrocytic tumors WHO grade II showed the highest probability for highly progressive disease (0.12).

A more favorable course of disease with no intervention at all or only one surgical intervention (states 1 and 2), i.e. non-progressive PLGG, was found for patients older than 1 year at diagnosis (1-7^11/12^ years: 0.54; ≥ 8 years: 0.69) and for tumor location in the cerebral hemispheres (0.77) or the cerebellum (0.76). Patients with a tumor in the lateral ventricles had the highest probability to stay stable without any adjuvant therapy (1.00). Five years after diagnosis patients with a tumor of non-astrocytic histology, i.e. neuronal and mixed neuronal-glial tumors as well as low-grade neuroepithelial or glial lesions not otherwise specified [LGG-NOS], had a probability of 0.75 and 0.69, respectively, to have had at most one surgical intervention (states 1 and 2). Still, the latter group of patients showed a probability of 0.06 for 2^nd^ adjuvant therapy and as well for death. Patients with radiologic tumor diagnosis and without histological confirmation were most likely to remain observed (0.57; state 1) or to receive one adjuvant therapy only (0.43, state 4). Sixty-five percent (n=198) of these tumors were located in the optic pathways and 58.7% (n=178) were patients with NF-1 (data not shown). This is reflected by the state-probabilities for NF-1 patients with main tumor location in the optic pathway (n=180; 77.3%; data not shown) not to be treated at all (0.36) or to have received only one adjuvant therapy (0.45) 5 years after diagnosis.

### 3.4 Impact of disease state at 2 years after diagnosis upon prognosis for the subsequent 3 years

Supplemental table S4 displays 3-year prediction and Aalen SE for further disease development for patients assessed at 2 years after diagnosis at their respective states. Figure 9 displays the corresponding state-probabilities with exact numbers for each state-probability at the time point 5 years after diagnosis. Patients who had received no intervention at all or surgical interventions without prior adjuvant therapy in the first 2 years after diagnosis (states 1 and 2) had the lowest probability (0.01-0.02) for highly progressive PLGG (states 5 and 6), while this 5-year-probability increased noticeably for patients who had undergone their 1^st^ adjuvant therapy (state 4) already in the first 2 years after diagnosis (0.25). The probability for death increased with the number of interventions that a patient had undergone in the first 2 years after diagnosis.

## 4. Discussion

In this study, we used a MSM to delineate a more refined course of PLGG disease in the German SIOP-LGG 2004 cohort of 1587 patients from diagnosis throughout possible subsequent interventions, i.e. surgery, ChT and RT. To our knowledge this is the first multi-state analysis in the field of neuro-oncology, especially so in PLGG disease. With the help of the MSM we were able to evaluate the probabilities for the evolution of 7 predefined states of disease and 3 levels of progressiveness - for the whole cohort as well as for defined subgroups. Based on the state of disease 2 years after diagnosis, we generated a prediction model for the subsequent 3-year disease development.

In our cohort the distribution of epidemiological characteristics such as sex, age at diagnosis, NF-status, tumor site, and histologic subgroups was comparable to other recent series^5^,^6^,^17^. Within a follow-up period of 6.3 years, patients underwent up to 5 surgical interventions and/or up to 5 adjuvant therapies, resulting into 22 intervention-states with 44 transitions.

At 5 years after diagnosis the highest probability for patients was either to have received no intervention at all (0.11) or one surgical intervention only (0.49). A relevant probability was to have undergone one adjuvant therapy (0.19) or at least two surgical interventions without adjuvant therapy (0.11), defined by us as low progressive disease. Still, the probability for needing more than one adjuvant treatment - defined as highly progressive PLGG - was 0.08. In the series of Gnekow et al., 2012 and Stokland et al., 2010 the comparable treatment groups comprised 1031 patients (n=668 observation arm including surgical interventions; n=363 with non-surgical intervention) and 639 patients (n=474 observation arm; n= 165 with non-surgical intervention), respectively. Due to the lack of similar analyses no direct comparison to treatment and risk groups of other cohorts can be made. While risk group definitions generally consider time and frequency of tumor-related events, in our model probabilities assessed by the MSM relate to interventions following tumor-related events. Nevertheless, we identified subgroups with a higher probability for the necessity of more than one adjuvant treatment, i.e. infants, tumor location in the SML, and patients with astrocytic tumors WHO grade II. We also revealed subgroups with fewer interventions - indicating a more favorable course of disease, i.e. older age (1-7^11/12^ years and ≥ 8 years), tumor location in the cerebral hemispheres, the cerebellum and the lateral ventricles, histology of neuronal and mixed neuronal-glial tumors or LGG-NOS, and patients with NF-1. The number of necessary interventions could be a surrogate parameter for more or less aggressive tumor biology.

In our cohort of patients with PLGG, the general course of disease was favorable with the highest probability to have no or only one surgical intervention and a low probability for death, i.e. non-progressive biology. This corresponds to previous findings of classical survival analyses of larger patient series reporting excellent OS in the range of 90% for various groups of PLGG, that even included subgroups with multiple and multimodal interventions^5^,^6^,^8^,^17^,^43^.

Still, we found a relevant probability for patients with PLGG to suffer from low (0.30) or highly progressive PLGG (0.08) 5 years after diagnosis. These probabilities indicate the burden of treatment for surviving patients. Both surgical interventions with the risk of permanent neurological impairment due to damage of healthy brain tissue^13^,^14^, as well as multiple adjuvant treatments with the risk for enhanced long-term neurotoxicity^3^, may lead to significant impairment of long-term quality of survival.

Few studies focus upon relapse or progression beyond first adjuvant therapy and efficacy of second-line treatment, with either small numbers^9^-^12^, selected subgroups of PLGGs, i.e. only astrocytoma grade I and II^3^, or surgery-only^7^. In addition to those limitations, results with respect to progression-free survival of those reports cannot be compared to state-probabilities due to the different concept of the MSM integrating the extensive course of disease of a large number of patients. The fact that diagnostic entities and underlying WHO criteria have changed over the last decades further limits comparability of our results to former studies.

The risk and protective factors identified in the German SIOP-LGG 2004 cohort when using the MSM confirmed the results of the classical clinical survival analysis. An impaired prognosis with a higher risk for treatment failure and progressive disease was reported for infants, location other than cerebellum especially the SML, and LGG WHO grade II^3^-^6^,^15^-^17^.

In our cohort, patients with a tumor location in the SML were characterized by a higher probability for more advanced disease states (0.17). Yet, they had also a high probability to stay untreated (0.22) within 5 years after diagnosis. This may reflect two subgroups of PLGG located within the visual pathway, i.e. on the one hand those associated with NF-1 and good prognosis and on the other hand sporadic visual-pathway glioma with a more aggressive growth behavior^17^,^44^,^45^.

Patients with a PLGG of neuronal and mixed neuronal-glial histology had an excellent prognosis, reflected in a low probability for highly progressive PLGG (0.04). At the same time, they showed a higher probability for either one (0.75) or several (0.12) surgical interventions without prior adjuvant therapy. This may relate to the distribution pattern of tumor location with an easier access for surgery, i.e. mainly in the cerebral hemispheres (69%, data not shown) and less frequent in the SML (8.4%, data not shown).

Finally, patients with LGG-NOS were difficult to analyze. The heterogeneity and histological uncertainty of this mix of PLGGs that do not meet clear criteria for histological classification hampered elaborating general statements for this group. Therefore, molecular biological analyses are urgently needed for clarification of these tumor entities and to subdivide histological subgroups^38^,^46^,^47^.

The MSM allowed predicting further outcome on the basis of the state of disease at 2 years after diagnosis: The more interventions were necessary in the first 2 years after diagnosis, the higher was the probability for further interventions as well as for death.

This finding might facilitate the assessment and estimation of disease course of future patients with PLGG and serve as a basis for clinical decision-making at different time points of an often-chronic disease.

## Limitations

We acknowledge certain limitations of our study. This study was purely explorative. Moreover, some subgroups could not be analyzed separately due to the small number of patients. We did not include extent of resection at 1^st^ surgery into subgroup analysis, as from a statistical point of view this is a time-dependent variable, with the future extent of resection not known at the time of diagnosis. For surgical intervention, we did not distinguish between therapeutic tumor resection and diagnostic biopsy. Due to the protocol's stratification structure of adjuvant therapy and the possibilities for physician's choice of further adjuvant intervention type, i.e. ChT or RT, we were not able to analyze influence of type of non-surgical intervention onto subsequent disease course. We acknowledge that especially the cohort of state 4 is inhomogeneous with respect to the variety of prior surgical interventions. It included patients who received no, one, two or more prior surgeries of different extent and at varying time points. For the sake of robust statistical analysis, we refrained from separating these subgroups further. Nevertheless, from a clinical point of view, this state reflects a group of patients whose tumor progression was more difficult to handle, as the decision for adjuvant therapy was made only when PD or threatening clinical symptoms could not be controlled with a surgical intervention. Due to a different statistical design of the MSM as well as to changing treatment strategies and WHO criteria over the last decades, our results could not be directly compared to other clinical studies of PLGG. Finally, as information on molecular markers was not available for patients of the SIOP-LGG 2004 study we could not include them into the histological subgroup analysis.

## Conclusion

In summary, we illustrated the potentials and feasibility of a MSM for PLGG, extending classic survival analysis and incorporating different states within the course of patients' disease, especially of chronic forms. Our study provides a comprehensive and refined picture of the heterogeneity of PLGG, and helps to describe the course of disease and treatment with respect to epidemiological subgroups over time. Our disease-state-probabilities reaffirm risk and prognostic factors already described, particularly regarding their relation to the risk for multiple interventions. The distribution of disease-states confirmed the known dichotomy of tumor behavior in the SML, i.e. some tumors needing no intervention at all, while others requiring multiple treatments. The prognostic model developed in this study provides the possibility for a better assessment of disease and may serve as a tool for clinical decision-making for patients with disease progression. Patients with non-progressive disease in the first 2 years after diagnosis are unlikely to show further need of intervention within the next 3 years while patients with progressive disease behavior in the first 2 years after diagnosis most likely further maintain this aggressive biology.

Further studies with an a priori multi-state design including molecular and toxicity data are necessary to confirm our findings and enable more exact and individualized treatment recommendation for less frequent subgroups. To distinguish subgroups further and better explain divergent growth behavior, biological markers need to be included for future risk group analysis^38^,^46^ - especially since there is the hypothesis of mainly stable molecular genetics in PLGG^48^ that might determine the growth of the individual tumor even in the long run. Molecular data will be considered and analyzed with a MSM in the ongoing LOGGIC Core Bioclinical Databank.

### Novelty and Impact

First multi-state analysis of a very large population-based cohort of pediatric low-grade glioma [PLGG]. It extends classical survival analysis by considering subsequent and competing events of chronic diseases, such as PLGG. Our seven-state model delineated a more refined course over time, including influence of subsequent events and prognostic factors. We identified three levels of progressiveness and generated a prediction model for PLGG-disease. This unique approach will be validated prospectively in the ongoing LOGGIC Core Bioclinical Databank.

## Supplementary Material

Supplementary figure and tables.

## Figures and Tables

**Table 1:** Demographic and clinical characteristics of German patients with pediatric low-grade glioma (n=1587)

	median	(min / max)
**Age at diagnosis [years]**	7.60	(0.10 / 17.90)
**Age-group at diagnosis [years]**	**total (n)**	**(%)**
<1	75	(4.7)
1-7^11/12^	748	(47.1)
≥ 8	764	(48.1)
**Sex**		
Female	740	(46.6)
Male	847	(53.4)
**Main location at diagnosis**		
Cerebral hemispheres	320	(20.2)
Supratentorial midline	580	(36.6)
Cerebellum	467	(29.4)
Caudal brain stem	119	(7.5)
Spinal cord	56	(3.5)
Lateral ventricles	44	(2.8)
Initially disseminated	1	(0.1)
**Main histology at diagnosis**		
Pilocytic Astrocytoma WHO I	869	(54.8)
Diffuse Astrocytoma WHO II	136	(8.6)
Oligodendroglioma WHO II	4	(0.3)
Oligoastrocytoma WHO II	8	(0.5)
Neuronal and mixed neuronal-glial tumors	203	(12.8)
Low-grade neuroepithelial or glial lesions not otherwise specified [LGG-NOS]	63	(4.0)
No histology/radiologic diagnosis-only	304	(19.2)
**Neurofibromatosis [NF] status**		
NF negative	1352	(85.2)
NF-1	233	(14.7)
NF-2	2	(0.1)
**Tuberous sclerosis complex [TSC]**		
TSC negative	1561	(98.4)
TSC positive*	26	(1.6)
**Extent of resection at 1^st^ surgery (n=1283)**		
Complete resection	522	(32.9)
Subtotal resection	138	(8.7)
Partial resection	360	(22.7)
Biopsy-only	263	(16.6)

1587 patients with pediatric low-grade glioma [PLGG] treated in Germany between 01.04.2004 and 31.03.2012 and followed until 27.04.2016 in the International Society of Paediatric Oncology - Low Grade Glioma subcommittee [SIOP-LGG] 2004 study. The percentage (%) of patients is given in brackets in respect to the whole group. * treatment with everolimus for subependymal giant cell astrocytoma in TSC patients was not documented, they remained in the observation group.

## Abbreviations

PLGG: Pediatric low-grade glioma
WHO: World Health Organization
OS: Overall survival
SML: Supratentorial midline
MSM: Multi-state model
MAPK: mitogen-activated protein kinase
SIOP-LGG: International Society of Pediatric Oncology - Low-Grade Glioma
NF-1: Neurofibromatosis type 1
ChT: Chemotherapy
RT: Radiotherapy
VC: Vincristine plus Carboplatin
VCE: Vincristine, Carboplatin plus Etoposide
NF-2: Neurofibromatosis type 2
TSC: Tuberous Sclerosis Complex
SE: standard error
LGG-NOS: Low-grade neuroepithelial or glial lesions not otherwise specified
SD: Stable disease, < 25% volume changes
PD: Progressive disease, ≥ 25% increase in tumor volume or new lesion
OR: Objective response, 25-50% reduction in volume
PR: Partial response, > 50% reduction in volume
