# Supplementary material for: Natural History of Pediatric Low-Grade Glioma Disease - First Multi-State Model Analysis
Source: J Cancer. 2019 Oct 17;10(25):6314–26. doi: 10.7150/jca.33463 (PMC6856735; doi:10.7150/jca.33463)
Supplement: Supplementary file 1 — Supplementary figure and tables. [file jcav10p6314s1.pdf]

Natural History of Pediatric Low-Grade Glioma Disease – First Multi-State Model Analysis

SUPPLEMENTALS

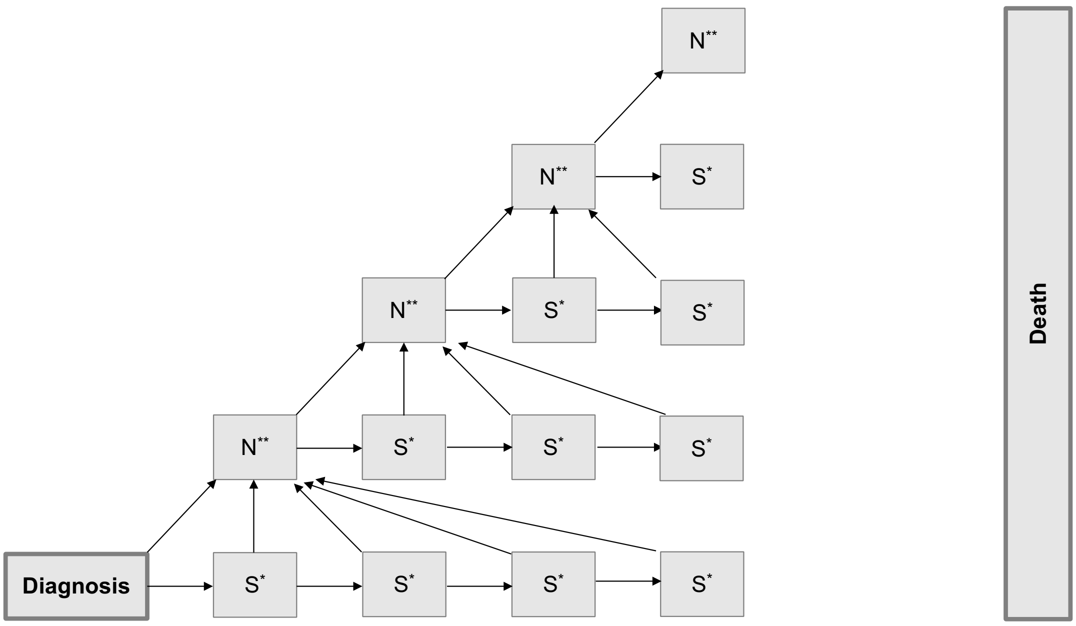

Supplemental figure S1: Schematic multi-state model for PLGG. Schematic model of possible states (boxes) and transitions (arrows) for disease development of PLGG. Starting state was defined as “diagnosis”, transient states as surgical intervention (S*) or non-surgical intervention (N**), i.e. chemotherapy or radiotherapy. Absorbing state “death” can be reached from all other states. For reasons of better clarity, arrows into the absorbing state have been omitted.

	State 1 [no intervention]	State 2 [1st surgery]	State 3 [≥2nd surgery]	State 4 [1st ChT/RT]	State 5 [surgery after 1st ChT/RT]	State 6 [≥2nd ChT/RT]	State 7 [Death]
Time point [years]	Probability (SE)	Probability (SE)	Probability (SE)	Probability (SE)	Probability (SE)	Probability (SE)	Probability (SE)
1	0.17 (0.009)	0.58 (0.012)	0.06 (0.006)	0.16 (0.009)	0.01 (0.002)	0.01 (0.003)	0.00 (0.002)
2	0.15 (0.009)	0.54 (0.013)	0.09 (0.007)	0.18 (0.010)	0.01 (0.002)	0.02 (0.004)	0.01 (0.003)
3	0.13 (0.008)	0.52 (0.013)	0.10 (0.008)	0.19 (0.010)	0.01 (0.002)	0.04 (0.005)	0.02 (0.004)
4	0.11 (0.008)	0.50 (0.013)	0.10 (0.008)	0.19 (0.010)	0.02 (0.003)	0.05 (0.005)	0.02 (0.004)
5	0.11 (0.008)	0.49 (0.013)	0.11 (0.008)	0.19 (0.010)	0.02 (0.004)	0.06 (0.006)	0.02 (0.004)
6	0.10 (0.008)	0.48 (0.013)	0.11 (0.008)	0.20 (0.011)	0.02 (0.004)	0.07 (0.007)	0.03 (0.004)
7	0.10 (0.008)	0.46 (0.013)	0.11 (0.009)	0.20 (0.011)	0.02 (0.004)	0.07 (0.007)	0.03 (0.005)
8	0.10 (0.008)	0.46 (0.014)	0.11 (0.009)	0.20 (0.012)	0.02 (0.004)	0.08 (0.008)	0.03 (0.005)
9	0.09 (0.009)	0.45 (0.016)	0.11 (0.011)	0.20 (0.013)	0.02 (0.005)	0.08 (0.008)	0.05 (0.008)
10	0.09 (0.009)	0.43 (0.019)	0.11 (0.014)	0.21 (0.017)	0.02 (0.005)	0.09 (0.009)	0.05 (0.009)

Supplemental table S2: State-probabilities and corresponding Aalen standard error [SE] for whole group of patients with PLGG at time points 1 to 10 years after diagnosis. State-probabilities and corresponding Aalen SE for the whole group of patients with PLGG (n=1587) at time point 1 to 10 years after diagnosis. State 1: no intervention. State 2: first surgery without prior adjuvant therapy. State 3: two or more surgeries without prior adjuvant therapy. State 4: first adjuvant therapy. State 5: one or more surgeries after first adjuvant therapy. State 6: two or more adjuvant therapies. State 7: death of any cause. Levels of progressiveness: non-progressive (states 1 and 2), low progressive (states 3 and 4), and highly progressive (states 5 and 6) PLGG. ChT: chemotherapy. RT: radiotherapy**.**

Subgroups of patients with PLGG	State 1 [no intervention] Probability (SE)	State 2 [1st surgery] Probability (SE)	State 3 [≥2nd surgery] Probability (SE)	State 4 [1st ChT/RT] Probability (SE)	State 5 [surgery after 1st ChT/RT] Probability (SE)	State 6 [≥2nd ChT/RT] Probability (SE)	State 7 [Death] Probability (SE)
**Age-group [years]**							
<1	0.16 (0.041)	0.19 (0.044)	0.11 (0.034)	0.16 (0.041)	0.03 (0.017)	0.27 (0.049)	0.09 (0.032)
1-7 11/12	0.13 (0.012)	0.41 (0.018)	0.10 (0.011)	0.25 (0.016)	0.02 (0.005)	0.07 (0.010)	0.02 (0.005)
≥ 8	0.09 (0.010)	0.60 (0.018)	0.11 (0.012)	0.14 (0.013)	0.02 (0.005)	0.03 (0.006)	0.02 (0.005)
**Sex**							
Female	0.11 (0.012)	0.46 (0.019)	0.12 (0.012)	0.20 (0.015)	0.02 (0.005)	0.06 (0.009)	0.02 (0.006)
Male	0.11 (0.011)	0.51 (0.018)	0.09 (0.010)	0.18 (0.014)	0.02 (0.005)	0.06 (0.008)	0.02 (0.005)
**Main location**							
Cerebral hemispheres	0.05 (0.013)	0.72 (0.027)	0.15 (0.021)	0.05 (0.013)	0.01 (0.006)	0.01 (0.005)	0.01 (0.006)
Supratentorial midline	0.22 (0.017)	0.18 (0.016)	0.04 (0.008)	0.35 (0.020)	0.04 (0.009)	0.13 (0.014)	0.04 (0.008)
Cerebellum	0.02 (0.006)	0.74 (0.021)	0.18 (0.019)	0.05 (0.011)	0.00 (0.002)	0.00 (0.003)	0.00 (0.002)
Caudal brain stem	0.10 (0.027)	0.36 (0.045)	0.05 (0.020)	0.33 (0.045)	0.00 (0.000)	0.12 (0.030)	0.05 (0.020)
Spinal cord	0.04 (0.022)	0.44 (0.065)	0.15 (0.048)	0.28 (0.059)	0.00 (0.000)	0.02 (0.019)	0.07 (0.034)
Lateral ventricles	0.15 (0.052)	0.74 (0.067)	0.10 (0.049)	0.00 (0.000)	0.00 (0.000)	0.00 (0.000)	0.00 (0.000)
**Main histology**							
Pilocytic astrocytoma I°	0.00 (0.002)	0.59 (0.017)	0.14 (0.012)	0.17 (0.013)	0.03 (0.006)	0.06 (0.008)	0.02 (0.004)
Diffuse astrocytoma II°	0.01 (0.006)	0.45 (0.043)	0.14 (0.031)	0.19 (0.035)	0.01 (0.006)	0.11 (0.026)	0.10 (0.025)
Neuronal and mixed neuronal-glial tumors	0.00 (0.000)	0.75 (0.031)	0.12 (0.024)	0.07 (0.019)	0.02 (0.010)	0.02 (0.010)	0.01 (0.007)
Low-grade neuroepithelial or glial lesions [LGG-NOS]	0.00 (0.000)	0.69 (0.058)	0.02 (0.014)	0.14 (0.043)	0.04 (0.022)	0.06 (0.028)	0.06 (0.030)
**Neurofibromatosis type 1 [NF-1]**							
NF-1 negative	0.06 (0.007)	0.56 (0.014)	0.12 (0.009)	0.15 (0.010)	0.02 (0.004)	0.06 (0.007)	0.03 (0.005)
NF-1 positive	0.36 (0.033)	0.06 (0.016)	0.02 (0.008)	0.45 (0.034)	0.02 (0.009)	0.09 (0.020)	0.00 (0.004)

Supplemental table S3: Five-year state-probabilities and corresponding Aalen standard error [SE] for subgroups of patients with PLGG. State-probabilities (State 1-7) and corresponding Aalen SE for relevant subgroups of patients with PLGG (≥ 40 patients per subgroup) at time point 5 years after diagnosis. State 1: no intervention. State 2: first surgery without prior adjuvant therapy. State 3: two or more surgeries without prior adjuvant therapy. State 4: first adjuvant therapy. State 5: one or more surgeries after first adjuvant therapy. State 6: two or more adjuvant therapies. State 7: death of any cause. Levels of progressiveness: non-progressive (states 1 and 2), low progressive (states 3 and 4), and highly progressive (states 5 and 6) PLGG. ChT: chemotherapy. RT: radiotherapy.

starting from	**State 1** [no intervention] Probability (SE)	**State 2** [1st surgery] Probability (SE)	**State 3** [≥2nd surgery] Probability (SE)	**State 4** [1st ChT/RT] Probability (SE)	**State 5** [surgery after 1st ChT/RT] Probability (SE)	**State 6** [≥2nd ChT/RT] Probability (SE)	**State 7** [Death] Probability (SE)
State 1	0.75 (0.030)	0.10 (0.020)	0.00 (0.001)	0.13 (0.021)	0.01 (0.002)	0.02 (0.004)	0.00 (0.000)
State 2		0.88 (0.012)	0.06 (0.009)	0.05 (0.007)	0.00 (0.001)	0.01 (0.001)	0.00 (0.002)
State 3			0.83 (0.031)	0.14 (0.026)	0.01 (0.002)	0.02 (0.004)	0.01 (0.008)
State 4				0.74 (0.025)	0.07 (0.013)	0.18 (0.021)	0.01 (0.006)
State 5					0.55 (0.104)	0.42 (0.094)	0.04 (0.018)
State 6						0.84 (0.049)	0.16 (0.049)

Supplemental table S4: Three-year prediction model for disease-state development starting from state 1-6 at 2 years after diagnosis. Five-year state-probabilities (State 1-7) and corresponding Aalen standard error [SE] for patients with PLGG having had reached state 1-6 at time point 2 years after diagnosis. State 1: no intervention. State 2: first surgery without prior adjuvant therapy. State 3: two or more surgeries without prior adjuvant therapy. State 4: first adjuvant therapy. State 5: one or more surgeries after first adjuvant therapy. State 6: two or more adjuvant therapies. State 7: death of any cause. Levels of progressiveness: non-progressive (states 1 and 2), low progressive (states 3 and 4), and highly progressive (states 5 and 6) PLGG. ChT: chemotherapy. RT: radiotherapy**.**
